# Morphologic Spectrum of Undetermined Causes of Hemoptysis- A Pathologist’s Role

**DOI:** 10.5146/tjpath.2020.01495

**Published:** 2020-09-15

**Authors:** Nidhya Ganesan, Umamaheswari Gurusamy

**Affiliations:** Department of Pathology, PSG Institute of Medical Sciences and Research, Tamilnadu, India

**Keywords:** Hemoptysis, Immunologic, Diffuse alveolar hemorrhage, Capillaritis

## Abstract

***Objective:*** Hemoptysis is the expectoration of blood or blood-streaked sputum from the tracheobronchial tree. The etiology may derive from nonneoplastic conditions such as infections, chronic pulmonary diseases, and vasculitis or neoplastic causes. Sometimes a definitive cause for hemoptysis cannot be found after ample diagnostic workup. The role of biopsy in such cases is to help the clinician in arriving at the final diagnosis. Diffuse alveolar hemorrhage is the main histopathological finding in hemoptysis and it appears with diffuse chest infiltrates radiologically.

***Material and Method:*** A retrospective study of 2 years duration was conducted to identify the morphological spectrum of diseases presenting with hemoptysis. A total of 243 lung biopsies obtained by various methods were retrieved in this study period and 20 cases with hemoptysis of undetermined etiology were detected.

***Results:*** Based on imaging and histopathology findings, the etiological causes of hemoptysis were divided into hemoptysis with and without capillaritis or due to tumor/tumor-like lesions and due to miscellaneous conditions. The most common etiology was vasculitis followed by infections.

***Conclusion:*** Histopathology helps to detect the etiology, particularly in cases of hemoptysis due to non-immunologic causes. In immunologic cases, histopathological findings may support the diagnosis in correlation with the clinical/imaging features.

## INTRODUCTION

Hemoptysis is the expectoration of blood from the pulmonary parenchyma or the tracheobronchial tree below the level of the glottis. The expectoration of blood can vary from a small amount of blood-streaking sputum to massive bleeding with life-threatening consequences. The clinical diagnostic algorithm should aim to differentiate and identify the etiology of hemoptysis, since massive hemoptysis may result in a fatal outcome (1,2).

The common causes of hemoptysis include pulmonary tuberculosis (TB), malignancy, bronchiectasis, chronic bronchitis, pneumonia, and fungal infections. The other rare causes are vasculitis due to collagen vascular diseases and hereditary hemorrhagic telangiectasia, essential cryoglobulinemia, Behcet’s disease, acute lung transplantation rejection, drug-induced (e.g., chemotherapeutic agents, propylthiouracil), idiopathic pulmonary hemosiderosis, and toxin or inhalation injury (3).

About 30% of cases attending respiratory clinics and presenting with hemoptysis pose a problem in clinical diagnosis, and the histopathology plays a vital role in diagnosing these conditions. The condition can be localized or diffuse depending upon the underlying etiology (4). Intra alveolar hemorrhage, fibrin deposition, and hemosiderin-laden macrophages associated with or without capillaritis are the common findings observed in cases of diffuse alveolar hemorrhage (DAH) (5). Radiologically, DAH shows patchy/ diffuse bilateral areas of lung consolidation mainly involving the lower lobes and perihilar regions (6).

A literature search revealed only a few reports on using histopathological interpretation in diagnosing unknown causes of hemoptysis. This is vital to diagnose and treat the condition earlier, as it prevents disease progression, and constituted the reason for conducting the current study. This study aims to identify the various uncommon aetiologies of hemoptysis based on histopathological features and the pathologist’s approach in diagnosing this condition.

## MATERIAL and METHODS

All lung biopsies, including lobectomy specimens received in the department of pathology, between Jan 2018 to Jan 2020 (2 years duration) were retrospectively reviewed. Of the received 243 specimens, 20 cases had hemoptysis with undetermined causes. The obtained specimens were derived from various procedures and included 117 transbronchial lung biopsies (TBLB), 62 endo bronchial lung biopsies (EBLB), 28 guided (Ultra sonogram /CT) biopsies, 19 thoracoscopic/Video-assisted (VATS) biopsies, and 17 lobectomies. The cases with a history of hemoptysis associated with common causes such as tuberculosis (TB), carcinoma, bronchitis, bronchiectasis, and interstitial lung diseases (ILD) were excluded from the study. The clinical data obtained were age, sex, presenting complaints, clinical diagnosis/differential diagnosis, and significantly associated co-morbidities. Laboratory investigations such as autoimmune workup (ANCA, ANA, etc.), sputum for culture & acid-fast bacilli (AFB), bronchoalveolar lavage (BAL) for culture and cytology, and other relevant information were collected. The findings of imaging (high-resolution computerized tomography - HRCT) and bronchoscopic findings were recorded if available (not always performed).

Ethical approval for this study was obtained from PSG Institute of Medical Sciences & Research (Approval number: 20/117, Date: 27.05.2020).

## RESULTS

Out of 243 lung biopsies received, a total of 20 cases with symptoms of hemoptysis due to undetermined etiology were included in our study.

### Histopathological Findings

Based on imaging and histopathological findings, the etiology of hemoptysis was divided into localized and diffuse/bilateral pulmonary involvement (Table 1). Depending upon the histological findings, the causes of hemoptysis were further divided into

- Hemoptysis with capillaritis (Table 2)

- Hemoptysis without capillaritis (Table 3)

- Unusual tumors and tumor-like lesions (Table 4)

- Miscellaneous uncommon causes

**Table 1:** Etiological classification based on imaging & histopathological findings.

**Causes of hemoptysis**			
**Localized**	**Number of cases**	**Diffuse /bilateral**	**Number of cases**
Pulmonary hamartoma	1	GPA	3
Aspergillosis	1	Silicosis	3
Myelolipoma	1	EGPA	2
Mucor	1	Invasive aspergillosis	1
Pulmonary endometriosis	1	Invasive mucor+aspergillosis	1
Mucoepidermoid carcinoma	1	Multiple metastases from a leiomyosarcoma	1
Probably benign mesenchymal neoplasm	1	PAM	1
		Pulmonary intravascular hemangioma	1

**GPA:** Granulomatosis with Polyangiitis / Wegener’s, **EGPA:** Eosinophilic granulomatosis with polyangiitis / Churg-Strauss syndrome, **PAM:** Pulmonary alveolar microlithiasis.

**Table 2:** Causes of hemoptysis with capillaritis.

	**Granulomatosis with Polyangiitis (GPA)**				**Eosinophilic granulomatosis with polyangiitis (EGPA)**	
	**Case - 1**	**Case-2**		**Case-3**	**Case 1**	**Case 2**
**Age, sex**	40, M	46, F		51, F	43, M	42, M
**Complaints**	Cough, hemoptysis, and dyspnoea					
**Serology**	Anti-CCP, PR3-ANCA&RF: positive		PR3-ANCA positive	PR3-ANCA positive	RF: positive	PR3-ANCA: weakly positive
**Imaging studies**	Bilateral nodules		Ground glass changes, calcified nodule	Bilateral consolidation with few nodules	Bilateral nodules	Bilateral nodular and cavitating lesions.
**Type of biopsy**	EBLB		CT guided biopsy	CT guided biopsy	TBLB	VATS lung biopsy
**HPE findings:** ** ** **Vascular changes:**	• Capillaritis, venulitis • Fibrinoid necrosis (Figure 2)		Vague outlines of thrombosed blood vessels	Vasculitis and Fibrin thrombi (Figure 2)	Eosinophilic infiltration of the vessel wall	• Necrosis of the vessel walls with inflammatory cells infiltration (Figure 3) • Organizing, recanalizing and fibrin thrombi
**Parenchymal changes**	• Granulomatous inflammation with MNG cells (Figure 2) • Interstitial inflammation		• Necrosis, karyorrhexis, hematoxyphilic nuclear dust • Neutrophilic micro abscess	• Necrosis, karyorrhectic debris • Granulomatous response with MNG cells • Neutrophilic micro abscess	• Eosinophilic microabscess • Palisading granuloma	• Intraalveolar hemorrhage (Figure 3) • Eosinophilic microabscess, parenchymal necrosis • Perl's stain highlights the hemosiderin-laden macrophages (Figure 4)

**MNG cells:** Multinucleate giant cells, **HPE:** Histopathological examination, **EBLB:** Endobronchial lung biopsy, **CT:** Computed tomography, **VATS:** Video-assisted thoracoscopic surgery, **TBLB:** Transbronchial lung biopsy.

**Table 3:** Causes of hemoptysis without capillaritis.

	**Silicosis**			**Fungal infection**				
	**Case -1**	**Case-2**	**Case-3**	**Case-1**	**Case-2**		**Case-3**	**Case-4**
**Age, sex**	61, M	54, M	58, M	28, M	56, M		45, F	15, F
**Complaints**	Cough, hemoptysis and dyspnoea.			Cough, hemoptysis, and dyspnoea				
**Another relevant history**	Exposure to silicon dust +			Post Koch’s sequelae		DM, CKD	Post TB, DM.	DM, DKA+, Mild PAH
**Imaging studies**	Bilateral lung nodules&hilar nodes	Calcified nodes	B/L nodules, calcification	NA		Cavitatory consolidation	B/L subpleural nodules	B/L cavitating consolidations
**Type of biopsy**	TBLB			Right upper lobe segmentectomy		EBLB	Right lower lobectomy	TBLB
**HPE** **Findings**	• Nodules of epithelioid and pigment laden histiocytes, predominantly around the vascular channels (Figure 5)			• Dilated bronchi forming a cavity containing the fungal ball • Tissue, angioinvasion & infarct • Fibrosis, chronic granulomatous inflammation • Oxalate crystals (Figure 6)				
**Special study**	Polarizer: birefringent crystals (Figure 5 inset)			GMS & PAS stains: septate, acute-angled branching hyphae of aspergillus		PAS & GMS stains: broad, aseptate and wide-angle branching hyphae of mucor	PAS & GMS stains: Tangles of fungal elements, consistent with aspergillus and mucor	

**DM:** Diabetes Mellitus, **CKD:** Chronic kidney disease, **TB:** Tuberculosis, **NA:** Not available, **DKA:** Diabetic ketoacidosis, **PAH:** Pulmonary artery hypertension, **B/L:** Bilateral, **HPE:** Histopathological examination, **EBLB:** Endobronchial lung biopsy, **TBLB:** Transbronchial lung biopsy, **GMS:** Grocott Methenamine Silver Stain.

**Table 4:** Unusual tumors and tumor-like lesions of the lung.

**Diagnosis**	**Pulmonary hamartoma (n=1)**	**High-grade Spindle cell sarcoma - metastatic LMS (n=1)**	**Benign mesenchymal neoplasm (n=1)**	**Pulmonary myelolipoma (n=1)**	**Endobronchial mucoepidermoid carcinoma, high grade (n=1)**
**Age, sex**	46, F	59, M	51, M	65, M	39, F
**Complaints**	Cough, hemoptysis, and dyspnoea				
**Imaging studies**	Solitary pulmonary nodule.	Nodular masses in both lungs - suggested metastases.	-	Endobronchial nodules -? TB/sarcoidosis	-
**Bronchoscopy**	NA	NA	Irregularisation in left lingula? a blood clot? growth	-	Left main bronchus tumor with a possible endobronchial carcinoid.
**Type of biopsy**	Lobectomy	EBLB	EBLB	EBLB	Left Pneumonectomy
**Gross**	Glistening lobulated mass of 3x2.5 cm	-	-	-	Endobronchial ulcerated solid, yellow-white polypoidal mass
**HPE** **findings**	Circumscribed lesion of mature cartilage interspersed by adipose tissue, smooth muscle, and fibrovascular tissue. Foci of ossification, chondromyxoid area.	• Pleomorphic spindle cells • Coagulative necrosis. Positive for SMA and desmin.	• Polypoidal lesion • Fascicles of spindle cells • Cellular myxoid stroma. • Express Vimentin and SMA	• Circumscribed lesion of thin trabecular bone enclosing adipocytes and hematopoietic elements • MPO-highlighting the myeloid cells (Figure 7).	• Infiltrating neoplasm, • Solid nests, cribriform pattern, and mucin filled acini. • Squamoid morules (Figure 8). • Positive for Pan CK and P63

**LMS:** Leiomyosarcoma, **NA:** Not available, **SMA:** Smooth muscle actin, **MPO:** Myeloperoxidase, **HPE:** Histopathological examination, **EBLB:** Endobronchial lung biopsy, **TB:** Tuberculosis.

**Figure 1:**
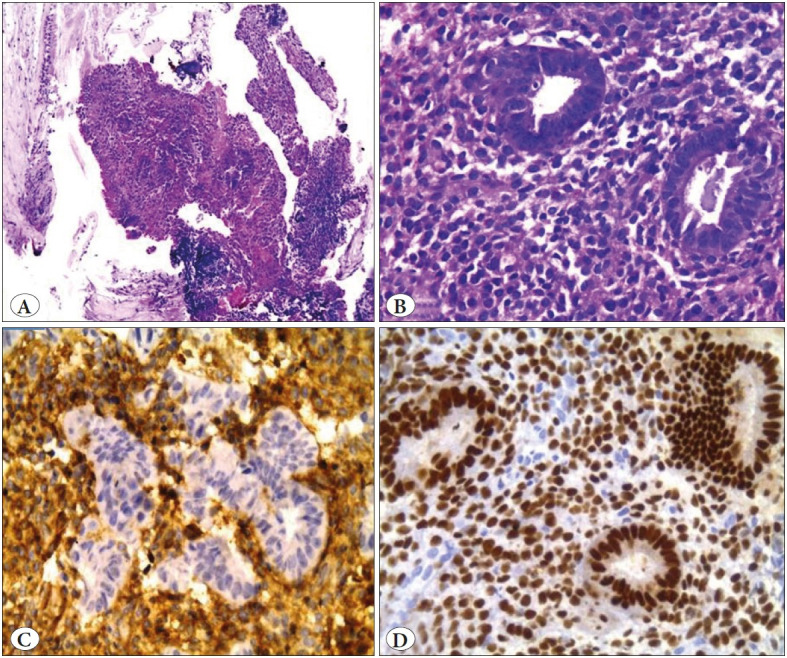
Bronchial endometriosis. **A)** Bronchial mucosa with a focus of endometrial glands and stroma (H&E; x4). **B)** High power view (H&E; x40). **C)** CD10 positive stromal cells (IHC; x40). **D)** ER positive epithelial & stromal cells (IHC; x40).

**Figure 2:**
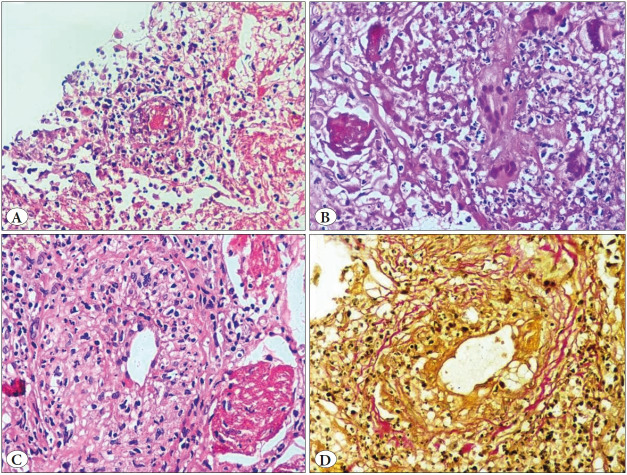
Granulomatosis with polyangiitis. **A)** Capillaritis with fibrin thrombi (H&E; x10). **B)** Scattered multinucleated giant cells, karyorrhectic debris & parenchymal necrosis (H&E; x40). **C)** Mediumsized vessel wall with neutrophilic infiltration and destruction (H&E; x10). **D)** Verhoeff-Van Gieson (VVG) stain highlights the outline of a vessel wall (VVG; x40).

**Figure 3:**
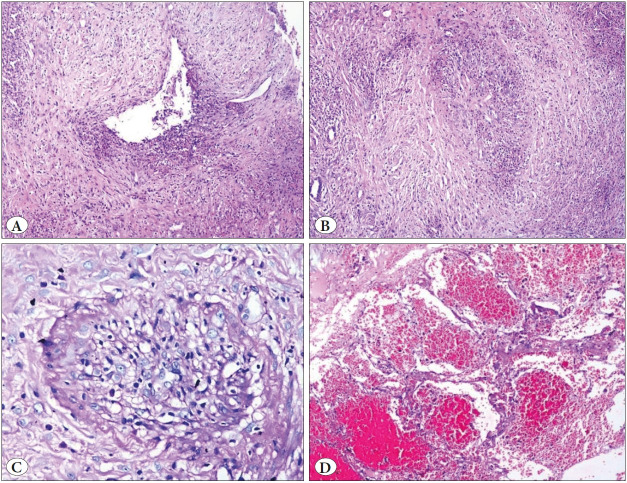
Eosinophilic granulomatosis with polyangiitis. **A-B)** A medium-sized artery infiltrated by eosinophils and histiocytes with partial destruction of a vessel wall (H&E; x10 & x20)**. C)** (PAS stain; x40). **D)** Intra-alveolar hemorrhage (H&E; x10).

**Figure 4:**
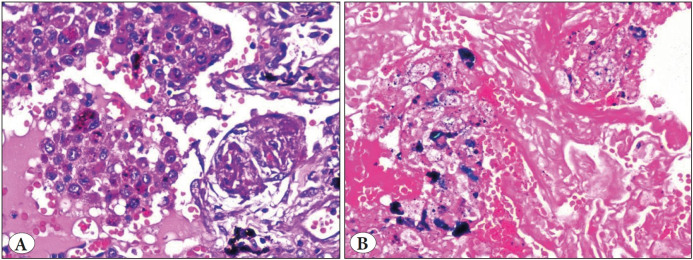
Hemosiderin-laden macrophages **A)** (H&E; x40). **B)** (Perl’s stain; x40).

**Figure 5:**
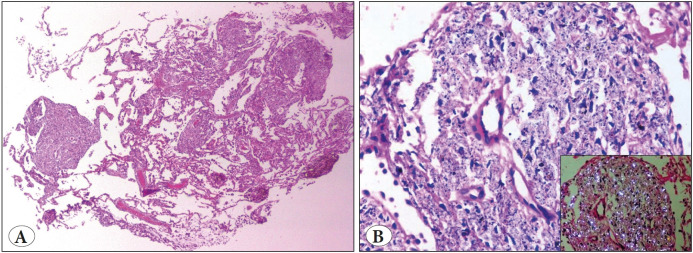
Silicosis. **A)** Multiple, patchy silicotic nodules (H&E; x10). **B)** Perivascular nodular collection of pigmented histiocytes (Inset-birefringent crystals under polarized light) (H&E; 40).

**Figure 6:**
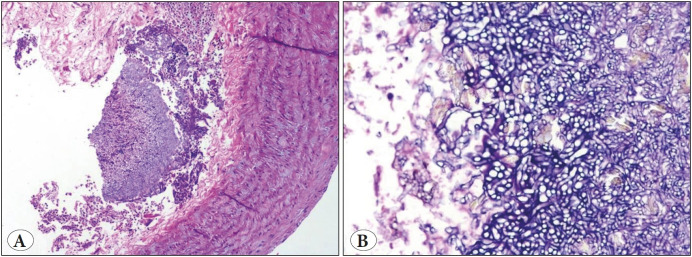
Fungal infection. **A)** Intravascular plugs of fungal organisms (H&E; x4). **B)** Septate, acute angle branching hyphal fungal organisms consistent with aspergillus with oxalate crystals (PAS; x10).

**Figure 7:**
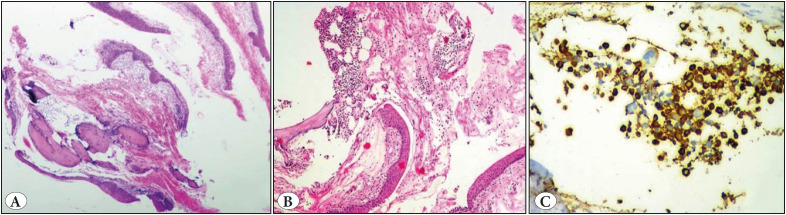
Myelolipoma. **A)** Bronchial mucosa with squamous metaplasia and a fairly circumscribed lesion composed of adipocytes admixed with hematopoietic elements (H&E; x4). **B)** High power view (H&E; x10). **C)** Myeloperoxidase stain (IHC; x10).

**Figure 8:**
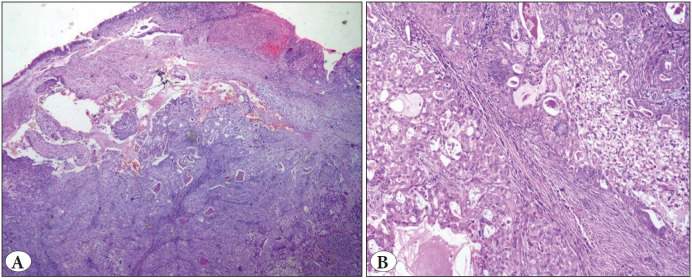
Endobronchial mucoepidermoid carcinoma. **A)** Showing admixture of squamous and glandular elements (H&E; x4). **B)** High power view (H&E; x10).

### Miscellaneous uncommon causes are listed below:

### Pulmonary Alveolar Microlithiasis (n=1)

HRCT of a 34-year-old male who presented with hemoptysis showed bilateral ground-glass densities with septal calcification -? Interstitial lung disease. TBLB sections showed distended alveoli containing concentric lamellated, calcific material with few hemosiderin-laden macrophages.

### Pulmonary Endometriosis (n=1)

A 43-year-old man with a history of cirrhosis secondary to hepatitis ‘C’ had recurrent episodes of hemoptysis. Bronchoscopy revealed a mucosal white patch in the left lingular segment. The biopsied lesion showed lung tissue intimately admixed with a focus of endometrial glands and stroma. CD 10 immunostaining of the stroma, as well as estrogen and progesterone receptor staining of the glandular component, were both consistent with endometriosis (Figure 1).

### Pulmonary Intravascular Haemangioma (n=1)

HRCT of an elderly male showed multiple small nodules in both the lungs and a lobulated enhancing soft tissue lesion attached to the anterior wall of the right atrium-? Malignancy. TBLB sections showed lung parenchyma with medium to large-sized vessels containing an intraluminal, adherent lesion of tubular vascular spaces lined by endothelial cells. CD31 & CD34 immunostaining highlighted the endothelial cells. Since the right atrial mass could not be biopsied, the clinical/imaging findings were correlated and the final HPE diagnosis made was intravascular capillary haemangioma/?fragmented from cardiac haemangioma.

## DISCUSSION

Haemoptysis is considered to be a serious medical condition and has a broad differential diagnosis. All cases with microscopic evidence of hemorrhage do not present with hemoptysis clinically. Similarly, all patients who presented with hemoptysis will not show alveolar hemorrhage histologically (4).

### Haemoptysis with Capillaritis (Granulomatosis with Polyangiitis & Eosinophilic Granulomatosis with Polyangiitis)

Pulmonary vasculitis is characterized by inflammation of the small and medium-sized vessel wall, causing pulmonary hemorrhage. Imaging studies show multiple lung cavitary nodules/masses, which may mimic metastasis. Also in some conditions, the lung infiltrates are transitory making the diagnosis a challenging one (7).

Schnabel et al. stated that histopathological findings may vary depending upon the type of biopsy. They found out that the yield of guided lung biopsy is high when compared to TBLB since gross lesions are easily visible. Similarly, most of our cases had undergone guided biopsies and were adequate (8).

Travis et al. suggested that capillaritis is a morphologic marker of DAH in their study. They concluded that capillaritis and necrotizing granulomatous inflammation with mixed inflammatory cell infiltrate were pathognomonic features of GPA. Parenchymal necrosis, extravasated fibrin, and scattered multinucleate giant cells in a biopsy could suggest the diagnosis of a GPA even in the absence of granuloma. The destruction of the vessel wall can be highlighted by VVG elastic stains. However, correct sampling is essential for the maximum yield (9,10).

The diagnosis of EGPA can be made if the biopsy shows granulomatous vasculitis with extravasated eosinophils and evidence of eosinophilic pneumonia (11). Correspondingly, three cases in our series showed capillaritis and the remaining two were diagnosed based on relevant clinical and imaging findings.

### Haemoptysis without Capillaritis

**Fungal Infection: **Alveolar hemorrhage resulting from fungal infection is infrequent. The possible pathogenesis of hemoptysis in fungal infection could be 1) due to chronic inflammation as the granulation tissue lining the cavity is rich in capillaries, 2) these new vessels are rather fragile and can rupture easily, and 3) erosion of the blood vessels adjacent to the cavity (12). The presence of calcium oxalate crystals in a patient with a cavitary lesion will raise suspicion of a fungal infection and the pathologist should search for the same in the adjacent area (13). Likewise, our cases also had similar histology which would have led to hemoptysis.

**Silicosis: **An autopsy case study by Kyeong et al. revealed that unlike silicosis which affects mainly the lung parenchyma, silico - tuberculosis also affects the pulmonary vasculature causing hemoptysis (14). Our cases did not have evidence of tuberculosis. Streak-type hemoptysis seen in our cases could be explained by the destruction of small-sized blood vessels by fibrosis and chronic inflammation (15).

### Unusual Tumors and Tumor-Like Lesions of the Lung

**Primary Pulmonary Mesenchymal Tumors: **Accounts for less than 1% of all lung malignancy. These tumors show lineage differentiation and can be diagnosed in the lung with similar histological and immunological criteria as in other sites (soft tissue) (16). Pulmonary hamartoma is the most common benign mesenchymal tumor of the lung, and is predominantly seen in older males**. **Geramizadeh et al. and Ahmed et al. in their study stated that the occurrence of hemoptysis in hamartoma is very rare and results from giant or endobronchial tumors causing erosion of the vessels (17,18). In contrast to their findings, our patient was a middle-aged female with a small peripherally located pulmonary hamartoma.

According to Hashimoto et al., it is important to identify and differentiate metastatic mesenchymal tumors correctly, since most mesenchymal tumors presented as occult/unknown primary with pulmonary metastatic foci (19). Identically, one of our cases had metastatic LMS with a known diagnosis of primary LMS of the thigh.

**Mucoepidermoid Carcinoma (MEC):** MEC is a rare salivary gland type endobronchial tumor with mucous secreting cells, squamous cells, and intermediate cells. A definitive diagnosis requires bronchoscopy and representative biopsy as this tumor exhibits morphological heterogeneity. It can be misdiagnosed as primary non-small cell lung carcinoma if the biopsy is inadequate (20,21). The diagnosis of this rare neoplasm was possible since ours was a pneumonectomy specimen.

**Myelolipoma:** Pulmonary myelolipoma is distinctly unusual and rarely presents with hemoptysis. Embolic origin, reticuloendothelial cell metaplasia, and proliferation of hematopoietic stem cells are possible mechanisms of histogenesis. Tumour arising adjacent to the bronchial cartilage may show bony trabeculae and this explains the possible histogenesis of the metaplastic theory (22). Our case also revealed a similar histology.

### Miscellaneous Uncommon Causes

**Pulmonary Endometriosis: **Pulmonary endometriosis is a rare form of extra pelvic endometriosis, occasionally reported in men. Cases of male endometriosis have typically been linked to increased circulating estrogen levels. Peripheral conversion of androstenedione and testosterone to the circulating estrogens was noted in men with cirrhosis of the liver (23). Our patient had a history of cirrhosis, possibly leading to an altered hormonal state that interacted with a reactive/metaplastic process.

**Pulmonary Alveolar Microlithiasis (PAM):** PAM is a rare autosomal recessive lung disease characterized by the accumulation of concentrically laminated calcospherites within the alveolar spaces. Lauta in his study stated that there is a striking clinico-radiologic disparity in this condition (24). Since our patient did not have other system involvement, the possibility of PAM over secondary calcification was favored by correlating the clinical and imaging features.

**Pulmonary Intravascular Haemangioma:** The co-existence of cardiac and pulmonary haemangioma is very rare and very few reports of such an association have been described so far. Imaging studies may help in diagnosing cardiac haemangioma while histopathology is essential for confirmation. Complete excision is the treatment of choice in resectable cardiac haemangioma and solitary pulmonary haemangioma (25). Since the general condition of the patient was poor, resection was not possible in our case. Fragmented emboli from a cardiac haemangioma could also be considered as a differential.

In conclusion, in immunologic causes of hemoptysis, histopathological findings may support the diagnosis in correlation with the clinical/imaging features. However, in cases of non-immunologic causes of hemoptysis, a histopathologic examination is mandatory over clinical/imaging studies. The cases presented here are incredibly rare diseases with an uncommon presentation. The diagnosis is often missed or delayed, due to a low index of suspicion. Histopathological examination is imperative in unexplained cases of hemoptysis as it plays a vital role in therapy decisions.

## CONFLICT of INTEREST

The authors declare no conflict of interest.

## FUNDING

The authors declared that this study has received no financial support.

## References

[ref-1] Haro Estarriol M., Vizcaya Sánchez M., Jiménez López J., Tornero Molina A. (2001). [Etiology of hemoptysis: Prospective analysis of 752 cases]. Rev Clin Esp.

[ref-2] Lee Bo Ram, Yu Jin Yeong, Ban Hee Jung, Oh In Jae, Kim Kyu Sik, Kwon Yong Soo, Kim Yu Il, Kim Young Chul, Lim Sung Chul (2012). Analysis of patients with hemoptysis in a tertiary referral hospital. Tuberc Respir Dis (Seoul).

[ref-3] Quadrelli Silvia, Dubinsky Diana, Solis Marco, Yucra Demelza, Hernández Marcos, Karlen Hugo, Brigante Alejandro (2017). Immune diffuse alveolar hemorrhage: Clinical presentation and outcome. Respir Med.

[ref-4] Colby T. V., Fukuoka J., Ewaskow S. P., Helmers R., Leslie K. O. (2001). Pathologic approach to pulmonary hemorrhage. Ann Diagn Pathol.

[ref-5] Park Moo Suk (2013). Diffuse alveolar hemorrhage. Tuberc Respir Dis (Seoul).

[ref-6] Castañer Eva, Alguersuari Anna, Gallardo Xavier, Andreu Marta, Pallardó Yolanda, Mata Josep Maria, Ramírez José (2010). When to suspect pulmonary vasculitis: radiologic and clinical clues. Radiographics.

[ref-7] Lynch JP, Leatherman JW, Fishman JA (1998). Alveolar hemorrhage syndromes. Pulmonary Diseases and Disorders.

[ref-8] Schnabel A., Holl-Ulrich K., Dalhoff K., Reuter M., Gross W. L. (1997). Efficacy of transbronchial biopsy in pulmonary vaculitides. Eur Respir J.

[ref-9] Travis W. D., Colby T. V., Lombard C., Carpenter H. A. (1990). A clinicopathologic study of 34 cases of diffuse pulmonary hemorrhage with lung biopsy confirmation. Am J Surg Pathol.

[ref-10] Travis W. D., Hoffman G. S., Leavitt R. Y., Pass H. I., Fauci A. S. (1991). Surgical pathology of the lung in Wegener's granulomatosis. Review of 87 open lung biopsies from 67 patients. Am J Surg Pathol.

[ref-11] Suster S, Moran CA, Epstein JI (2013). Biopsy interpretation series. Biopsy interpretation of lung.

[ref-12] Corrin B, Nicholson AG (2011). Pathology of lungs.

[ref-13] Mukhopadhyay Sanjay, Erika D (2016). Nonneoplastic pulmonary pathology: An algorithmic approach to histologic findings in the lung.

[ref-14] Kyeong Hee Eun, Cheong Harin, Kim Hyoung Joong, Choi Young Shik (2012). Pulmonary hemorrhage with progressive massive fibrosis in a silicosis patient: An autopsy case. Korean J Leg Med.

[ref-15] Anna-Luise A, Katzenstein MD, David DA (2016). Diagnostic Atlas of Non-Neoplastic Lung Disease: A Practical Guide for Surgical Pathologists.

[ref-16] Travis WD, Brambilla E, Burke AP, Marx A, Nicholson AG (2015). WHO Classification of Tumours of the Lung, Pleura, Thymus, and Heart.

[ref-17] Geramizadeh Bita, Mottavvas Maedeh, Zeyaian Bijan, Amirian Armin (2019). Giant hamartoma of lung presented with massive hemoptysis: A rare case report and review of the literature. Rare Tumors.

[ref-18] Ahmed Saman, Arshad Ayesha, Mador M. Jeffery (2017). Endobronchial hamartoma; a rare structural cause of chronic cough. Respir Med Case Rep.

[ref-19] Hashimoto Hirotsugu, Tsugeno Yuta, Sugita Keisuke, Inamura Kentaro (2019). Mesenchymal tumors of the lung: diagnostic pathology, molecular pathogenesis, and identified biomarkers. J Thorac Dis.

[ref-20] Toit-Prinsloo Lorraine, Bunn Belinda K. (2016). Massive hemoptysis due to primary mucoepidermoid carcinoma of the lung in a 12-year-old. Forensic Sci Med Pathol.

[ref-21] El-Sameed Yaser Abu, Al Marzooqi Saeed H. (2012). Primary mucoepidermoid carcinoma of the lung. J Bronchology Interv Pulmonol.

[ref-22] Sato Katsuaki, Ueda Yoshimichi, Katsuda Shogo, Tsuchihara Katsuma (2007). Myelolipoma of the lung: a case report and brief review. J Clin Pathol.

[ref-23] Jabr Fadi I., Mani Venk (2014). An unusual cause of abdominal pain in a male patient: Endometriosis. Avicenna J Med.

[ref-24] Lauta Vito Michele (2003). Pulmonary alveolar microlithiasis: an overview of clinical and pathological features together with possible therapies. Respir Med.

[ref-25] Wang Chunping, Chen Hao, Sun Lin, Mei Yunqing (2017). Cardiac Cavernous Hemangioma Coexisting With Pulmonary Cavernous Hemangiomas and Giant Hepatic Hemangioma. Ann Thorac Surg.

